# Reflections After ACC.23 From the Council of the Women in Cardiology

**DOI:** 10.1016/j.jaccas.2023.101966

**Published:** 2023-08-03

**Authors:** Estefania Oliveros, Sarah Rosanel, Kristen Brown, Jennifer Co-Vu, Gina Lundberg, Kamala Tamirisa

**Affiliations:** a Division of Cardiovascular Disease, Department of Medicine, Temple University Hospital, Philadelphia, Pennsylvania, USA; b Memorial Regional Hospital, Miami, Florida, USA; c University of Nebraska Medical Center, Omaha, Nebraska, USA; d University of Florida Congenital Heart Center, Gainesville, Florida, USA; e Division of Cardiology, Department of Medicine, Emory University School of Medicine, Atlanta, Georgia, USA; f Texas Cardiac Arrhythmia, Dallas, Texas, USA

**Keywords:** cardiology, diversity and inclusion, women

**Figure:**
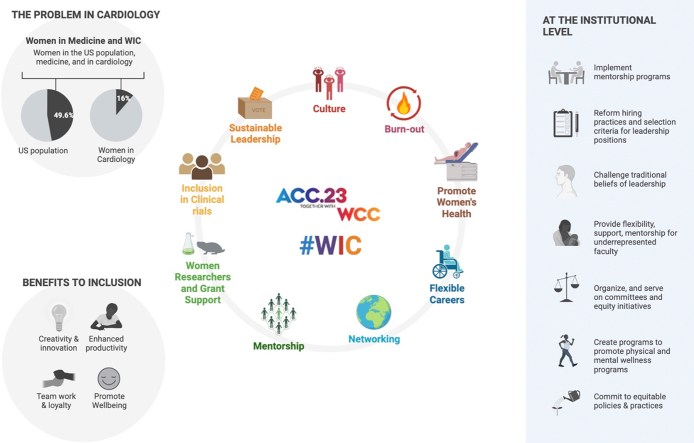

The American College of Cardiology (ACC) Women in Cardiology (WIC) Leadership Council hosted several sessions in the WIC Lounge at the ACC.23 Annual Meeting with the intention of collaborative discussion toward widening the pathway and furthering the opportunities to include WIC. Some sessions focused on empowering women, supporting leadership and career advancement, protecting women's health, and addressing the barriers in the work environment. Among the ACC members, the United States and international physician WIC members constitute approximately 15% according to the ACC member data in 2023. The purpose of this article is to share the efforts of WIC at the ACC.23 meeting and the actionable items discussed at these sessions toward a leveled playground for WIC and to create a safe space for open dialogue (Figure 1).

**Figure 1:**
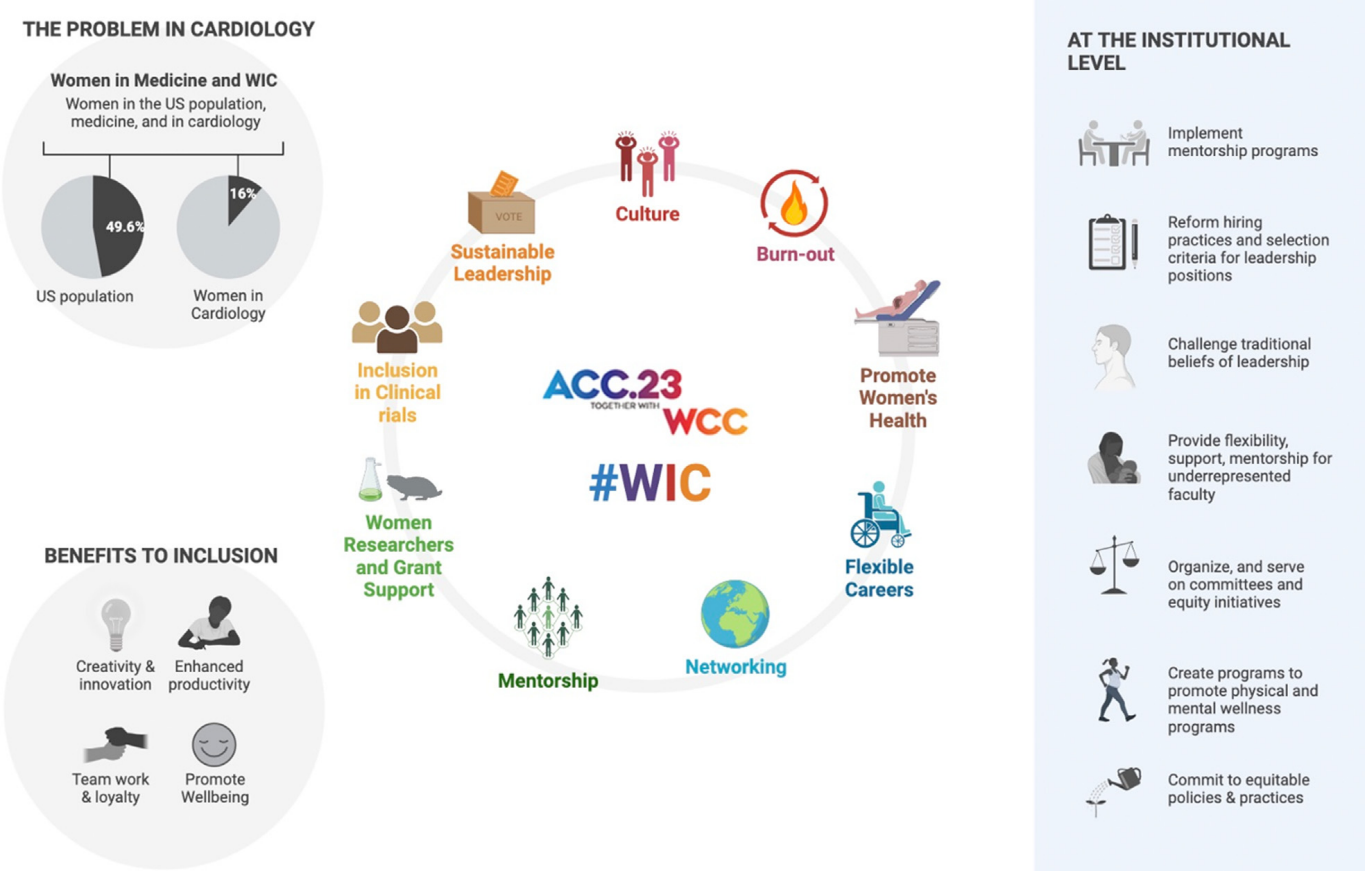
Women in Cardiology: Issues to Ideas.

*Created with Biorender.*

## ACC Women Leaders Session

### Moderator: Sharonne Hayes, MD

#### Panel and Speakers: Athena Poppas, MD, Claire Duvernoy, MD, Mary Norine Walsh, MD, Dipti Itchhaporia, MD

The WIC Section meeting launched with an empowering session with the ACC-WIC leaders. Even though women are underrepresented in leadership roles in cardiology, we were honored by the presence and willingness of the esteemed leaders who shared their experiences and pearls. The panel included 3 ACC past presidents and a board of trustees member. Their inspiring pathways showcased that women have made strides in ACC leadership. They shared their barriers with imposter syndrome, importance of self-advocating, and their success and failures. This session offered an honest discussion with potential solutions to overcome obstacles and how to be an effective leader by choosing professionalism, hard work, patience, and resilience.

### Ideas discussed

• Collective leadership that champions other WIC (and men)
• Building trust, fostering individual confidence, honesty, and integrity
• Defining clear goals with delegation
• Leveraging team members’ talents
• Being a good listener
• Creating a culture of “feedback loops” in conversations
• Developing a culture of adaptability and accountability and not losing the sight of the “big picture”
• Acting against implicit biases that undermine leadership expansion
• Diversifying teams to encourage varied ideas and talents

## Where Are the Women?

### Chair: Kamala Tamirisa, MD

#### Panel and Speakers: Doreen Defaria Yeh, MD, Janet K. Han, MD, Martha Gulati, MD, C. Michael Gibson, MD

Despite the number of WIC increasing over time, they are less likely to be full professors, receive research funding, have a registered clinical trial, and they are underrepresented on the editorial boards of cardiovascular journals. Women hold few departmental leadership positions in medicine, even in female-dominated specialties. Women authors of manuscripts studying these disparities presented their work and stimulated a lively discussion of where we are and what the future holds. Dr Defaria Yeh highlighted the sex differences in faculty rank among academic cardiologists in the United States; Dr Gulati offered data on gender differences in guideline authorships in the United States, Canada, and Europe; Dr Gibson offered solutions to end gender inequality in clinical trial leadership; and Dr Han discussed pathways to promote diversity and in inclusion in cardiovascular societies. An engaging discussion left the audience with some potential solutions as we look at the future.

### Potential solutions

• Deliberate sponsorship of women
• Intentional recruitment of WIC talent
• Engaging male allies
• Increasing efforts to empower WIC researchers with designated and paid time for research
• Offering support and resources
• Awarding grants to women

These initiatives need broad, intentional, and collective efforts at institutional, individual, and organizational levels.

## Increase WIC in Research

### Chairs: Kristen Brown, MD, Jennifer Rymer, MD

#### Panel and Speakers: Roxana Mehran, MD, Puja Mehta, MD, LaPrincess Cerise Brewer, MD, Tracy Wang, MD, Daniel R. Anderson, MD, Jennifer Rymer, MD

Research for academic advancement, personal satisfaction, and the science community’s betterment remains outside easy reach for many WIC today. The reasons for this sex disparity have been proposed and investigated, including the underrepresentation of women in the field and the lower likelihood of women to gain leadership positions,^1^ the lesser compensation,^2^ and underrepresentation in randomized trials.^3^

The benefits of having women researchers in original research within the cardiology field are endless. Research studies conducted by women primary investigators are more likely to include sex-based analyses. Models should be implemented to exemplify an accessible-distributed research team model to help increase women physician representation and improve the academic women cardiologists’ landscape. Dr Mehran explained the landscape of WIC in research and her pathway to becoming a successful clinical trialist, Dr Mehta described how to launch a pathway in research at the training and early career stages, Dr Brewer shared how to become funded by creating community-based research and to find ways to diversify the teams, Dr Wang highlighted her experiences as a successful trialist, Dr Anderson explained how to be an ally, and Dr Rymer offered research resources available to WIC.

### Highlighted solutions

• Building early mentorship programs for clinician scientists
• Incorporating basic, translational, community-based, and clinical outcomes research into early education
• Sponsoring industry-supported research
• Creating courses and support for effective grant writing

## Conversations Around Pregnancy Issues

### Chairs: Sarah Rosanel MD, Estefania Oliveros MD

#### Panel and Speakers: Jennifer Co-Vu, MD, Gina Lundberg, MD, Kamala P. Tamirisa, MD, Garima Sharma, MD, Toniya Singh, MD, L. Zahedi-Spung, MD

The decision by the Supreme Court in the case of Dobbs v Jackson and its impact on maternal health, including maternal and fetal cardiovascular health, was discussed. The American College of Obstetrics and Gynecology (ACOG) condemned this ruling, and currently the treatment of patients is dependent on the state governments’ rulings and terms. ACC also released a statement sharing similar sentiments with ACOG and that the College is deeply concerned about the potential implications of the Supreme Court decision regarding Roe v Wade on the ability of patients and clinicians to engage in shared discussions about maternal health, given the alarming maternal health crisis in the United States. This session included a talk by a well-recognized maternal and fetal medicine/high-risk obstetric specialist, Dr Zahedi-Spung, who educated the audience about the legal influence on clinical practice patterns across academic and private practice entities. The cardiologists’ professional role in the care of mothers and fetuses with heart disease, and how the legislation will affect our careers professionally, were addressed.

### Key points

• Know your state laws
• Advocate for improvement in laws in states where there are bans
• Monitor morbidity and mortality boards
• Watch for impact on women at the greatest risk for complications resulting from social determinants of health such as low income and limited education

## Flexible Schedules in Cardiology Careers

### Chairs: Gina Lundberg, MD, Toniya Singh

#### Panelists: Mary Norine Walsh, MD, Claire Duvernoy, MD, Sharonne Hayes, MD

A career is “an occupation undertaken for a significant period of a person's life and with opportunities for progress.: Therefore, throughout an individual’s life and career, many events, planned or unplanned, may occur that require flexibility, alterations, and changes from the system and the individual to accommodate a cardiologist’s career. There may also be additional roles or responsibilities that the cardiologist decides to embark upon, creating the need to adjust work-related duties or compensation models. The recent 2022 ACC Health Policy Statement on Career Flexibility in Cardiology provided the mainstem for the discussion.^4^ This statement is extensive and all-encompassing, with issues including parental leave, medical leave, changes in career, activities outside career, aging, and retirement. This statement affects fellows, early career, mid-career, and advanced career physicians of both sexes and all areas of cardiology careers, from academic to private practice, research, and industry.

### Key purposes of the statement

• All cardiologists would benefit from flexibility in work hours and responsibilities
• Help retain cardiologists who need time off temporarily and increase their longevity
• Provide flexibility to address the projected shortage of cardiologists

## Future

The ACC WIC Leadership Council objectives are to increase WIC in training, in the workforce, and in leadership while enhancing professionalism. WIC supports women of all ages, stages, and subspecialties. Fostering work-life integration as well as providing mentoring and networking opportunities while promoting personal well-being are essential. This year’s sessions were heavily attended not only by the WIC section members but also by male allies, ACC leadership, and other section members. By sharing the key points of these sessions, we hope to help highlight the ACC’s and ACC WIC’s efforts toward equity in cardiology and serve as a resource for our members, medical societies, and institutions.

## Funding Support and Author Disclosures

The authors have reported that they have no relationships relevant to the contents of this paper to disclose.
